# Neurodegenerative Disease and the Experience of Homelessness

**DOI:** 10.3389/fneur.2020.562218

**Published:** 2021-01-13

**Authors:** Stefanie Danielle Piña-Escudero, Lucía López, Sandeepa Sriram, Erika Mariana Longoria Ibarrola, Bruce Miller, Serggio Lanata

**Affiliations:** ^1^Global Brain Health Institute, University of California, San Francisco, San Francisco, CA, United States; ^2^Global Brain Health Institute, Trinity College, Dublin, Ireland; ^3^Department of Neurology, Memory and Aging Center, UCSF Weill Institute for Neurosciences, University of California, San Francisco, San Francisco, CA, United States

**Keywords:** homeless persons, frontotemporal dementia, neurodegenerative diseases, social determinants of health, underserved populations, Alzheimer disease, dementia, homelessness

## Abstract

**Introduction:** Today, half of the American homeless population is older than 50 years of age. This shift in age distribution among people experiencing homelessness has challenged our long-held views of the causes of homelessness. Age-related neurological diseases, especially neurodegenerative diseases of the brain (NDDB), may play a role eliciting homelessness in a significant proportion of vulnerable older adults. This article aims to explore relationships between homelessness and NDDB in a cohort of research participants enrolled in observational studies on NDDB at an academic center.

**Methods:** We reviewed charts of the Memory and Aging Center (MAC) of the University of California, San Francisco's database searching for research participants with NDDB that had direct relationship to homelessness. We reviewed all research visits conducted between 2004 and 2018 (*N* = 5,300). Research participants who had any relationship to homelessness were included in this analysis. NDDB was diagnosed via comprehensive neurological, functional, neuropsychological, and biomarker assessments. Non-parametric tests were used for analysis. Thirteen participants were found to have a direct relationship with homelessness. Seven were female and the median of education was 16 (IR: 12.0–19.5) years. Participants were divided into two groups: Those who experienced homelessness while symptomatic from a NDDB but before formal diagnosis (*n* = 5, Group 1); and participants with formally diagnosed NDDB who exhibited a new propensity toward homelessness (*n* = 8, Group 2). Compared to Group 2, participants in Group 1 were younger (*p* = 0.021) and showed similar results in the neuropsychological evaluation. In both groups, the most prevalent diagnosis was frontotemporal dementia. In Group 1, the majority of participants became homeless in the setting of a fragile socioeconomic situation and informants believed that NDDB contributed or caused their homeless state. In Group 2, a new propensity toward homelessness became manifest in different ways and it stood out that all of these participants were well-supported by family and friends during their illness.

**Conclusions and Relevance:** This case series highlights the role that NDDB may have in precipitating homelessness among vulnerable older adults, particularly in the setting of challenging socioeconomic circumstances and unsupportive living environments. Social ramifications of these findings, particularly pertaining to challenges around rehousing these individuals is discussed.

## Introduction

Every year, on a single night in January, the Department of Housing and Urban Development (HUD) conducts a national count of individuals experiencing homelessness in the U.S.—the Homelessness Point-in-Time Count. In 2019, roughly 567,715 Americans were homeless, and approximately half of them were older than 50 years (1). Indeed, whereas in previous generations the homeless population consisted mostly of young and middle-aged adults, the last three decades have seen a rapid increase in the prevalence of older homeless adults.

The rapid shift in the age distribution of our nation's homeless population has challenged our long-held views of the causes of homelessness. For decades, accepted narratives of homelessness were largely centered on the notion that homeless individuals embody a history of harsh childhood experiences and early-life circumstances that diminished their chances of educational and occupational attainment, which placed them at high risk of substance use and/or psychiatric disease, thus leading to increased risk of homelessness (2, 3). This view, however, has been recently challenged by the fact that the majority of older adults experiencing homelessness do not carry a history of preceding childhood abuse or trauma, mental illness, or drug use (4, 5). Instead, the life histories of older homeless adults tend to be less tumultuous than those who become homeless at a younger age, and the causes of homelessness among older adults point to more immediate life challenges, most often pertaining to socioeconomic stressors occurring in the setting of inadequate family/community support and a weak social safety net (6).

Our current national homelessness situation may be further exacerbated by the fact that a rising proportion of homed older adults live with mild or undiagnosed neurocognitive impairment caused by an underlying neurodegenerative disease of the brain (NDDB). These older adults may find themselves at a significantly increased risk of homelessness, especially when confronted with extraordinary socioeconomic stressors in the setting of unsupportive social environments. In this study, we aim to explore this idea by retrospectively analyzing all relationships to homelessness in a large cohort of research participants with NDDB in an academic center, as a secondary aim, we will explore if there are any cognitive, psychiatric/behavioral, and functional differences between the group that experienced homelessness and the group that exhibited a new propensity toward it.

## Methods

We conducted a chart review of the University of California, San Francisco (UCSF), Memory and Aging Center (MAC) research database. We searched for research participants diagnosed with NDDB that had any direct relationship to homelessness. We hypothesized that neurocognitive impairment, including impairments in social cognition, can contribute to homelessness by rendering individuals incapable of successfully overcoming unforeseen life challenges, particularly in the setting of inadequately supportive social environments.

The MAC conducts several large longitudinal observational research projects that follow persons with different forms of NDDB, including Alzheimer disease (AD) and frontotemporal lobar degeneration (FTLD). MAC research cohorts include asymptomatic and symptomatic individuals with familial/genetic forms of NDDB, as well as individuals with sporadic NDDB. All research participants undergo annual or biannual research evaluations consisting of comprehensive neurocognitive assessments that include detailed medical and neurological interviews, neurological and neuropsychological examinations, and blood, cerebrospinal fluid, and imaging biomarker studies. Participants also undergo detailed functional assessments using the Clinical Dementia Rating Scale. All the data is securely stored in a searchable electronic database.

For this manuscript, we searched all research visits conducted at the MAC between 2004 and 2018 (*N* = 5,300) using the following search terms, which were selected to identify research participants who had any relationship with homelessness: “home,” “homeless,” “street,” “service,” “services,” “car,” “crime,” “beggar,” and “shelter.”

Variables were described using frequency and proportion or median and interquartile range (IR). We used Fisher Exact Tests for categorical data and non-parametric tests for continuous variables. All statistical tests were calculated at the 0.05 level and 95% confidence intervals (CI). Analysis was performed using SPSS software for iOS (SPSS Inc., Chicago, IL, version 21.0).

## Results

We identified 13 research participants who had a direct relationship to homelessness. After reviewing each participant's specific relationship to homelessness, we divided the cohort into two groups: Participants who experienced homelessness while symptomatic from a NDDB, but before receiving a specific diagnosis of NDDB (*n* = 5, group 1); and participants who exhibited a new affinity toward homelessness after being diagnosed with a NDDB (*n* = 8, group 2). The median age of the 13 research participants included in this case series was 59 years (IR: 4.5–73.0). Half of all participants were female. The median level of education was 16 years (IR: 12.0–19.5).

Table 1 lists the cognitive, psychiatric/behavioral, and functional profiles of research participants within each of the two groups, based on data collected during each participant's first evaluation at the MAC. When comparing both groups, the only statistically significant difference that was found, was that participants in Group 1 are younger compared to their counterparts in Group 2 (*p* = 0.021). Nevertheless, both groups exhibited poor scores on the Mini-Mental State Exam, memory, language, visuospatial, frontal-executive tasks, and social cognition.

**Table 1:** Sociodemographic and neurocognitive characteristics of cases.

	**All (*N* = 13)**	**Group 1 (*n* = 5)**	**Group 2 (*n* = 8)**
Age	59 (54.5–73.0)	55 (50.0–57.0)	67.0 (59.7–79.5)
Female sex, No. (%)	7 (54.8)	4 (57.1)	3 (42.9)
Years of education	16 (12.0–19.5)	12.0 (11.5–15.5)	18.5 (13.7–20.0)
CDR total	2.0 (1.2–2.7)	2.0 (1.2–3.0)	2.0 (0.8–2.3)
MMSE	23.0 (15.5–29.0)	15.0 (0.0–29.5)	25.5 (18.2–29.5)
**Memory function**			
California verbal learning test short form 10-min recall (/9)	3.0 (0.0–7.0)	0.0 (0.0–7.0)	4.5 (0.2–8.2)
Benson figure copy 10-min recall (/17)	13.0 (7.5–14.0)	0.0 (0.0–12.0)	7.0 (1.0–12.0)
**Language function**			
Boston naming test spontaneous correct (/15)	12.0 (7.0–14.5)	8.0 (0.0–14.0)	13.0 (10.5–14.7)
**Visuospatial function**			
Benson figure copy (/17)	5.0 (0.0–5.0)	4.0 (0.0–4.0)	14 (13.0–15.5)
Calculations (/5)	3.0 (1.5–4.0)	2.0 (0.0–4.0)	3.5 (2.0–4.7)
**Frontal-executive function (attention, processing speed, working memory, executive functions)**			
Digits backward	3.0 (1.0–4.5)	2.0 (0.0–4.0)	3.5 (2.2–4.7)
Modified trails (correct lines per minute)	5.0 (0.0–14.0)	6.0 (3.0–6.0)	14.0 (5.0–14.0)
Stroop color naming (# correct in 60 s)	29.9 (0.0–38.5)	0.0 (0.0–42.5)	32.0 (13.7–36.0)
Stroop inhibition (# correct in 60 s)	9.0 (0.0–22.0)	0.0 (0.0–42.5)	10.5 (2.0–23.2)
Phonemic fluency (# correct in 60 s)	5.0 (0.5–7.5)	3.0 (0.0–5.5)	6.0 (1.5–12.5)
Semantic fluency (# correct in 60 s)	6.0 (1.0–16.0)	5.0 (0.0–15.0)	7.0 (2.7–17.0)
Design fluency filled dots (# correct in 60 s)	3.0 (0.5–6.5)	2.0 (0.0–10.5)	5.0 (1.2–7.2)
Design fluency repetitions	0.0 (0.0–2.0)	0.0 (0.0–10.5)	0.0 (0.0–1.5)
**Mood and social cognition**			
Geriatric Depression Scale	8.0 (4.0–13.5)	8.0 (4.0–14.0)	8.0 (4.0–14.0)
Number of behavioral signs and social cognition deficits observed during bedside evaluation (agitation, stimulus-boundedness, perseverations, decreased initiation, stereotypies, distractibility, loss of social engagement, impulsivity, social inappropriateness, fluctuating arousal)	2.0 (0.5–5.0)	2.0 (0.5–3.5)	2.0 (0.5–3.5)

*Means (m) and standard deviations (SD) are listed for each group unless otherwise noted*.

Table 2 shows the characteristics of the five MAC research participants who experienced homelessness while symptomatic from a NDDB, but before receiving a diagnosis (Group 1). Three of these participants received a diagnosis of frontotemporal dementia (FTD), and exhibited primarily frontotemporal atrophy on brain MRI, predominately right-sided. All participants exhibited behavioral signs and symptoms early in their disease course. Four of the five participants experienced neurocognitive decline in the setting of a fragile socioeconomic situation, and informants (close family or friends) believed that these participants' neurocognitive decline contributed or caused their homeless state. Notably, three of the five participants were evaluated by a health professional while homeless, and their neurocognitive symptoms and homeless state were reportedly attributed to substance abuse and/or a psychiatric disease, not neurodegenerative disease. Moreover, two participants were arrested for misdemeanors, and they were reportedly unable to properly adapt to jail settings, due to FTD. None of the participants in Group 1 were able to resume independent living after experiencing homelessness.

Table 2Participants who experienced a relation with homelessness.

**Table:** 

**Participant**	**Research diagnosis**	**Diagnostic biomarkers**	**Age range at onset of first signs and symptoms**	**Age range at diagnosis**	**Early neurocognitive changes (including changes in social cognition)**	**Age at onset of homelessness, duration of homelessness (in years)**	**Pertinent narratives obtained from research visit summary**
**GROUP 1. PARTICIPANTS WHO EXPERIENCED HOMELESSNESS PRIOR TO RECEIVING A DIAGNOSIS OF NEURODEGENERATIVE DISEASE**							
1	EOAD	*Brian MRI:* Cortical atrophy of the dorsal frontal and parietal lobes, with mild hippocampal atrophy. *Brain autopsy:* Alzheimer's Disease neurodegeneration.	38–40	47–49	*First symptoms:* Change in personality, behavior. *Symptoms during illness:* Irritability, social withdrawal, episodic memory deficits, word finding difficulties.	40–43, 3–4	The participant became divorced and unemployed concurrently to onset of cognitive decline. The participant subsequently became homeless. For years, his/her symptoms were attributed to other causes rather than to a neurodegenerative disease. When his/her parents took him/her back into their house, the disease had progressed to the extent the participant could no longer live independently.
2	svPPA	*Brain MRI: N/A* *Brain autopsy:* Frontotemporal lobar degeneration with tau-immuno-reactive inclusions (FTLD-tau) due to MAPT mutation.	unknown	55–57	*First symptoms:* Change in personality, behavior (burglary, alcohol abuse). *Symptoms during illness:* Semantic memory deficits, deficits in judgment, and problem-solving.	53–55, unknown	The participant became homeless shortly after becoming symptomatic. He/she remained homeless until arrested for burglary and was admitted to a mental health facility, where he/she received a diagnosis of adult-onset schizophrenia. At the hospital, he/she was observed looking through trash bins, taking food and cigarettes from other residents, removing his/her clothes and going to other resident's beds, and wandering aimlessly.
3	nfvPPA	*Brain MRI:* Severe, diffuse atrophy, mostly involving the frontal lobes and most pronounced on the right hemisphere. *Brain autopsy:* Frontotemporal lobar degeneration with TDP-immuno-reactive inclusions (FTLD-TDP), type A.	46–48	51–53	*First symptoms:* Change in personality, behavior (burglary, drug abuse). *Symptoms during illness:* Apathy, disinhibition, obsessive-compulsive behavior, mental rigidity, decreased speech output.	35–42, 7	The participant struggled to take care of his/herself due to cognitive decline. The participant lived with different people who took her/him into their homes until he/she lost his/her job and became homeless. While homeless, he/she was incarcerated after committing several minor misdemeanors associated with fixed and obsessive ideas about a specific singer. In prison, he/she was diagnosed with schizophrenia and was released to a group home where he/she exhibited disruptive behaviors that led to his/her being discharged to live with his/her daughter.
4	Probable bvFTD	*Brain MRI:* Bifrontal atrophy (orbitofrontal, mesial, and dorsolateral cortex), most notable on the right, as well as anterior insular and mild anterior temporal atrophy. *Brain autopsy:* N/A	52–54	57–59	*First symptoms:* Change in personality, behavior (disinhibition, drug abuse). *Symptoms during illness:* Deficits in judgment and problem-solving, word finding difficulties, attention, apathy, disinhibition, irritability.	51–52, 2	The participant became homeless after spending all his/her savings with his/her new partner. He/she ended up living with his/her brother, who described him/her as exhibiting new obsessive-compulsive behaviors and overeating. He/she also stole his prescription medications.
5	PSP	*Brain MRI:* Normal for age. *Amyloid PET (PIB): Negative* *Brain autopsy:* N/A	53	55	*First symptom:* Emotional lability, change in personality (depressed mood, irritability, anxiety). *Symptoms during illness:* word pronunciation difficulties.	35, 1	She became homeless after divorcing her husband. While homeless, she had “an awakening” experience, which she described as a period of “total peace and an absence of fear.” She experienced difficulty controlling her emotions since this experience.

**Table:** 

**Participant**	**Research diagnosis**	**Diagnostic biomarkers**	**Age at onset of first signs and symptoms**	**Age at Diagnosis**	**Main neurocognitive changes (including changes in social cognition)**	**Pertinent narratives obtained from research visit summaries**
**GROUP 2. Participants WHO EXHIBITED A NEW AFFINITY TOWARD HOMELESSNESS AFTER RECEIVING A DIAGNOSIS OF NEURODEGENERATIVE DISEASE**						
1	nfvPPA	*Brain MRI:* Diffuse dorsal frontoparietal atrophy, most pronounced on the left. Left medial temporal atrophy. *Amyloid PET (PIB): Negative* *Brain autopsy:* Frontotemporal lobar degeneration with tau immunoreactive inclusions (FTLD-tau), PSP.	73–75	74–76	*First symptoms:* Language dysfunction (word pronunciation difficulties). *Symptoms during illness:* Decreased speech output, swallowing difficulties.	Despite the participant's progressive language difficulties, he/she remained very active in his/her church and began volunteering at a women's homeless shelter.
2	nfvPPA	*Brain MRI:* Diffuse dorsal frontoparietal cortical atrophy (slightly worse on the left), with thinning of the corpus callosum. *Brain autopsy:* N/A	76–78	83–85	*First symptoms:* Language dysfunction (word pronunciation difficulties). *Symptoms during illness:* Worsening word finding difficulties, effortful speech output.	The participant became involved in feeding the homeless once a week through his/her church. He/she began volunteering for a project that helped dogs for the blind by doing everything that other people did not want to do.
3	svPPA	*Brain MRI:* Severe atrophy of the bilateral anterior temporal lobes, left greater than right. Moderate atrophy of the left amygdala and mild atrophy of bilateral medial frontal cortices and bilateral hippocampi, left more prominent than right. *Brain autopsy:* N/A	51–53	61–63	*First symptoms:* Language dysfunction (anomia) *Symptoms during illness:* Planning, organizing and multitasking difficulties, emotional lability.	Whereas in the past the participant was not seemingly bothered by homelessness, during his/her illness he/she was seen to be moved to tears when he/she saw a homeless person on the street.
4	Definite bvFTD	*Brain MRI:* Atrophy of the bilateral orbitofrontal cortex. Atrophy of the bilateral caudate nuclei, and less prominent hippocampal atrophy bilaterally. *Brain autopsy:* Frontotemporal lobar degeneration with TDP-43 immunoreactive inclusions (FTLD-TDP), Type B, with motor neuron disease	41–43	60–62	*First symptoms:* Change in personality, behavior (social withdrawal, loss of empathy). *Symptoms during illness:* Compulsive behaviors, disinhibition, impulsivity.	The participant had a strict schedule that included collecting food for the homeless once a week.
5	Probable bvFTD	*Brain MRI:* Bifrontal atrophy, right greater than left. *Brain autopsy:* N/A	56–58	59–61	*First symptoms:* Change in personality, behavior (restless, compulsive, speech stereotypy, ritualistic behaviors). *Symptoms during illness:* Depressive symptoms, irritability, disinhibition, hyperorality.	Despite not being wealthy, the participant donated considerable amounts of money to homeless people in the streets. He/she quit smoking and started going up to strangers trying to convince them not to smoke
6	Definite bvFTD-ALS	*Brain MRI:* Mild bilateral frontal lobe atrophy predominantly in the orbitofrontal cortex, dorsomedial frontal cortex, insular cortex, and anterior corpus callosum. *Brain 18 F-FDG PET CT:* Severe hypometabolism at frontal and prefrontal cortices bilaterally and moderate hypometabolism at anterior temporal cortices bilaterally. The parietal cortex and occipital cortex were preserved. *Brain autopsy:* Frontotemporal lobar degeneration and amyotrophic lateral sclerosis with TDP-43 immunoreactive inclusions (FTLD-TDP), Type B	52–54	58–60	*First symptoms:* Change in personality, behavior (apathy, loss of empathy) *Symptoms during illness:* Disinhibition, impaired judgment, delusions, compulsive behaviors, muscle twitching, balance difficulties, dysphagia.	The participant became very talkative with strangers and homeless people.
7	Probable bvFTD	*Brain MRI:* Mild frontal atrophy, mostly right sided, as well as anterior temporal, dorsoparietal, and anterior insular atrophy bilaterally. *Amyloid PET (AV45): Negative*	64–66	72–74	*First symptoms:* Change in personality, behavior (social withdrawal). *Symptoms during illness:* Impaired judgement, change in eating habits, apathy, loss of empathy, bilateral arm action tremor, generalized seizures	When the participant learned that his/her caregiver and her son were homeless, he invited them to come live at his/her partner's house without consulting with his/her partner.
8	Probable bvFTD	*Brain MRI:* Severe frontal, anterior temporal, and insular atrophy, most pronounced on the right. The hippocampal formations were also diminished in volume. *Brain autopsy:* N/A	49–51	53–55	*First symptoms: Change in personality, behavior (apathy)*. *Symptoms during illness:* Impaired judgement, disinhibition, change in eating habits, loss of empathy, planning and organizing difficulties	The participant developed a new tendency to tip large amounts of money to homeless people or street performers.

*nfPPA, non-fluent variant of primary progressive aphasia; EOAD, Early onset Alzheimer's disease; svPPA, Semantic variant of primary progressive aphasia; bvFTD, behavioral variant of frontotemporal dementia; MRI, Magnetic Resonance Image; 18 F-FDG PET CT, (18)F-fluorodeoxyglucose molecular imaging with positron emission tomography*.

Table 2 also shows the characteristics of the eight MAC research participants who exhibited a new propensity toward homelessness after being diagnosed with a NDDB (Group 2). All received a diagnosis of FTD, either behavioral variant frontotemporal dementia (bvFTD) or primary progressive aphasia (PPA). Participants with bvFTD had a frontotemporal pattern of cerebral atrophy on MRI, and exhibited deficits in frontal-executive tasks as well as social cognition. The participants with PPA showed early language deficits. Participants demonstrated a new propensity toward homelessness in different ways, such as becoming involved in helping homeless people through religious organizations, expressing unusual concern toward the precarious situation of homeless individuals, or giving money and/or food to homeless individuals while exhibiting little empathy for close family members. All of these participants were well-supported by family and friends during their illness. This new and unexpected interest in homeless individuals persisted despite placing some of these participants at risk for exploitation or physical risk.

## Discussion and Future Directions

Findings from this study support our hypothesis that cognitive and behavioral impairment resulting from early NDDB may add to the vulnerability of older adults struggling to maintain their independence; and when such impairment occurs in conjunction with challenging socioeconomic circumstances and unsupportive social environments, it may place them at increased risk of homelessness. Indeed, we identified a group of research participants with NDDB who experienced homelessness while symptomatic, but prior to receiving a formal diagnosis of NDDB (Table 2, Group 1), and our analysis of all available data from these participants strongly suggests that NDDB contributed to homelessness in all of them, and that socioeconomic stress and poor social support, further pushed these individuals to homelessness. To illustrate this point, we highlight the case of a participant with autopsy-confirmed frontotemporal lobar degeneration with TDP type A inclusions who early in his/her illness exhibited personality changes marked by disinhibition and apathy (Third participant of Group 1, Table 2). These changes were not promptly recognized as signs of neurologic disease, hence proper medical care was delayed. The participant became unable to work despite seemingly preserved neurocognitive function across other domains. He/she eventually became homeless. His/her erratic behaviors led to subsequent imprisonment and placement in a group home, but at this point the participant's neurocognitive disorder was such that he/she was unable to successfully navigate the daily routines of the group home setting. Upon reviewing this participant's chart, it appears that he/she would have remained homeless if not for the support of his/her daughter, who relatively late in the illness pursued medical care for the participant, leading to a diagnosis of frontotemporal dementia.

In order to better contextualize the significance of these findings it is important to highlight that the initial signs and symptoms of a specific NDDB (Alzheimer disease, frontotemporal lobar degeneration, etc.) are dictated by the brain networks that are primarily affected by the respective neuropathologic process (7). Beyond impairments in well-recognized cognitive domains (memory, visuospatial function, language, etc.), the first signs and symptoms of some forms of NDDB, especially those that involve primarily the frontal and anterior temporal lobes, often lead to deficits in social cognition marked by changes in socioemotional processing, personality, and behavior, whereas other cognitive domains remains relatively intact in early disease stages. Over the course of years, all persons affected by a NDDB progress from a mostly focal prodromal or mild illness to a more global and severe illness known as dementia, broadly defined as neurocognitive impairment that is severe enough to impede independent living. These two key aspects of the natural history of these diseases—their focal onset and long durations of illness—may be relevant to homelessness risk, as all participants in our study who became homeless prior to receiving a diagnosis of NDDB were in the early stages of illness (i.e., they were not demented) and exhibited focal deficits in social cognition and/or frontal-executive functions. Furthermore, it is noteworthy that 60% of all of these participants received a formal diagnosis of FTD, either the behavioral variant of FTD (bvFTD) or the language variant, also known as primary progressive aphasia (PPA). Persons with early FTD exhibit significant changes in social cognition and/or frontal-executive function, and/or language function, all of which can severely disrupt interpersonal relationships and corrode the affected person's social life, often in stigmatizing ways. Moreover, the behavioral and psychopathological changes observed in persons with early FTD are commonly misinterpreted as manifestations of a primary psychiatric disease, sometimes for many years before a neurodegenerative disease is suspected (8), thus adding to the social stigma and exclusion often experienced by those affected by this disease. Therefore, it is not surprising that in our study those participants who became homeless prior to receiving a diagnosis of NDDB were thought to be struggling from substance abuse and/or psychiatric illness by family members and/or healthcare providers, misconceptions that contributed to a delay in neurological evaluation.

Taken together, our analysis of these cases (Table 2, Group 1) leads us to conclude that the stigmatizing changes in socioemotional functioning, personality, and behavior observed in persons with FTD, coupled with deficits in frontal-executive and/or language function seen in these persons, may render them uniquely unable to overcome challenging life events, thus leading to homelessness even when their clinical presentations do not meet criteria for dementia.

Our study findings also suggest that the risk of homelessness in persons with early FTD is highest when the disease occurs in the setting of socioeconomic hardship and/or lack of robust family or community support, which is an increasingly common reality in the San Francisco Bay Area and in many other metropolitan regions of the U.S. Indeed, a rapidly increasing number of older adults in the Bay Area face unprecedented socioeconomic stress driven primarily by rising costs of living, neighborhood gentrification, social isolation, and chronic loneliness. Based on our experience caring for older adults with NDDB from diverse neighborhoods in the Bay Area, inadequate family support is common, as increasingly the children of older adults leave the area in search of more affordable places to live and raise a family. This is perhaps the main reason why in 2016 ~30% of older adults in San Francisco were living alone (9).

Findings in this study also highlight possible relationships between perceived criminal behaviors and homelessness among persons with NDDB. Previous studies have shown that persons with NDDB, especially those with FTD-spectrum disease, may engage in behaviors judged as criminal by our society and legal system; behaviors ranging from urinating in public, driving a vehicle while intoxicated, stealing, trespassing, and inappropriate sexual behaviors (10). Two participants in our study were arrested for misdemeanors while homeless, and they were reportedly unable to properly adapt to prison settings due to their neurocognitive deficits. This observation sheds light on the potentially significant utility of conducting comprehensive neurological and neurocognitive evaluations in imprisoned older adults, a significant proportion of which may have a NDDB and therefore require a different judicial system that is responsive to their needs.

The second group of participants included this study (Table 2, Group 2) exhibited a new propensity toward homelessness after being diagnosed with a NDDB. Of note, all of these participants received a diagnosis of FTD. We recognize three main behavioral phenotypes within this group. The first phenotype was observed in participants with a diagnosis of PPA-sv, who developed new emotionally-driven affinity for homeless people. Strong social-emotional responses have been observed in persons with PPA-sv, a phenomenon probably associated to damage of left ventral striatal networks, which may lead to increased social-emotional activity via upregulation of right-sided ventral networks (11); likewise, degeneration of the left orbitofrontal cortex in PPA-sv has been associated with increased agreeableness and increased interpersonal connection through positive emotion (11). The second phenotype was observed in participants with PPA-nfv, who exhibited propensity toward homelessness through new involvement in religious organizations. Previous research has shown that hypometabolism in the left middle and superior prefrontal cortex and left anterior superior temporal pole is associated with enhanced religiosity and charitable behaviors (12), which may explain this behavior. Finally, the third phenotype observed in our case series pertains persons with bvFTD, who demonstrated a new propensity toward helping homeless people during their illness. On the surface, this behavior appears to contradict one of the hallmark clinical manifestations of bvFTD, which is lack of empathy. The neuroanatomy of empathy, however, is complex, and recent models emphasize two interdependent mechanisms underlying empathic behavior (13). The first involves affect sharing—a person's cognitive-emotional ability to internally experience the emotional state of others; the second involves prosocial motivation—the desire to engage in helping behaviors. Studies have shown that persons with early-stage bvFTD can experience a loss of affect sharing while the frontal lobe anatomy driving prosocial motivation remains preserved (14). This scenario would explain how a person with early bvFTD can engage in the “right” behavior, such as feeding the homeless, but such behavior is not necessarily linked with a cognitive-emotional empathic state. In contrast to the first group, participants in this second group had a strong social network that might have prevented them from becoming homeless.

Finally, it is important to highlight that challenging social environments may be playing a major role in bringing upon homelessness in patients with NDDB, since no specific cognitive, psychiatric/behavioral or functional differences were found between the group that experienced homelessness (Group 1) and the group that exhibited propensity toward it (Group 2). In support of this idea, a recent study showed that a family member affected by FTD produces a mean decrease in household income from $75,000 to $99,000 12 months before diagnosis, to $50,000 to $59,999 12 months after diagnosis (15). Thus, supporting the notion that individuals with NDDB without an adequate social network can be easily driven into homelessness.

There are important limitations to our study. First, recruitment of research participants within our center is strongly biased toward highly educated and affluent individuals who have robust family support (a study partner, preferably a family member, is required for participation) and are actively receiving medical care. Therefore, our research cohorts certainly do not represent the true prevalence of homelessness among older adults with NDDB, which is probably significantly higher than that suggested by our findings. Moreover, the protective socioeconomic and highly supportive family backgrounds of our study participants may explain why we did not identify older adults who became homeless after receiving a diagnosis of NDDB. Based on our clinical and research experience, we believe that what prevents persons with early FTD from becoming homeless is the unwavering support of family and close friends. The question remains as to how many of our research participants with FTD would be homeless or marginally housed, even in early or mild stages of disease, if they were economically vulnerable and did not have a strong family support system—probably many. Furthermore, we suspect that many of the participants with bvFTD in this study who experienced a propensity toward homelessness after diagnosis would probably become homeless themselves as their disease progressed if they didn't have family members looking over them. Viewed differently, our study findings also raise the possibility that the prevalence of FTD in the general population is higher than previously recognized, as many persons with FTD may find themselves in a position in which they are less likely to be evaluated by specialists experienced in FTD diagnosis.

In order to elucidate more accurately the potential associations between NDDB and homelessness among older adults, longitudinal research studies that seek to characterize the neurocognitive health of homeless older adults using comprehensive and detailed behavioral neurology research procedures are needed. Shedding light on the role of NDDB in precipitating homelessness among vulnerable older adults could inform health care systems and other homelessness support systems tasked with determining the most effective and sustainable intervention for these persons. Indeed, at a structural societal level, we cannot expect those who have lost capacity to live independently due to NDDB to obtain the necessary resources and meet the necessary requirements to find and maintain housing without new modes of support that consider these neurodegenerative diseases.

More research is needed to explore the hypothesis that neurocognitive impairment that localizes to frontotemporal networks confers a higher risk of homelessness compared to other neurocognitive profiles, such as amnestic- or visuospatial-predominant ones. The fact that most of the participants included in this study received a diagnosis of FTD may not be merely coincidental. Indeed, deficits in attention, concentration, problem-solving, judgement, and/or multitasking, coupled with changes in social cognition, can be particularly disabling in society, especially among those who are still living largely independently and trying to adjust or adapt to a major life event. Indeed, frontal lobe dysfunction, particularly executive dysfunction, has been associated with homelessness in populations living in Australia (16) and Japan (17).

Moving forward, we will pursue community-based behavioral neurological studies of older homeless adults in both San Francisco and Oakland. We will collaborate with investigators of the HOPE-HOME study in Oakland, a longitudinal study of health outcomes of older adults experiencing homelessness, to conduct on-site comprehensive neurocognitive evaluations on homeless older adults, including detailed behavioral neurology examinations, cognitive examinations, and biomarker studies when possible. We will also partner with other community centers in San Francisco that serve older adults experiencing homelessness in order to conduct similar observational research studies. We believe this effort will shed light on specific neurocognitive diseases that may be contributing to homelessness. We believe that results from such studies could lead to important changes in state and governmental policies currently in place to assist homelessness older adults. Currently, these policies are mostly geared to address homelessness related to treatable diseases or conditions, not homelessness related to progressive and irreversible diseases of the brain. Shedding light on the role of NDDB in homeless older adults could powerfully inform health care systems and other support systems tasked with determining the most effective and sustainable interventions for these individuals.

## Data Availability Statement

The datasets presented in this article are not readily available because these data are currently part of other research projects. They are not available for sharing until the completion of all projects in which the subjects are participating. Requests to access the datasets should be directed to Serggio Lanata, serggio.lanata@ucsf.edu.

## Ethics Statement

The studies involving human participants were reviewed and approved by University of California, San Francisco, USA. The patients/participants provided their written informed consent to participate in this study.

## Author Contributions

SP-E, LL, SS, EL, BM, and SL participated in the original idea, data collection, and writing of the final article. All authors contributed to the article and approved the submitted version.

## Conflict of Interest

The authors declare that the research was conducted in the absence of any commercial or financial relationships that could be construed as a potential conflict of interest.
